# Hybrid Argon Plasma Coagulation for Barrett’s Esophagus and for Colonic Mucosal Resection—A Systematic Review and Meta-Analysis

**DOI:** 10.3390/biomedicines11041139

**Published:** 2023-04-10

**Authors:** Maria Manuela Estevinho, Rolando Pinho, João Carlos Silva, João Correia, Pedro Mesquita, Teresa Freitas

**Affiliations:** 1 Department of Gastroenterology, Vila Nova de Gaia Espinho Hospital Center, 4400-129 Vila Nova de Gaia, Portugal; rpinho@chvng.min-saude.pt (R.P.);; 2 Unit of Pharmacology and Therapeutics, Department of Biomedicine, Faculty of Medicine, University of Porto, 4200-450 Porto, Portugal

**Keywords:** Barrett’s esophagus, colon polyps, endoscopic mucosal resection, therapeutic endoscopy

## Abstract

Hybrid argon plasma coagulation (hAPC) is a novel technique that combines conventional argon plasma coagulation and waterjet submucosal expansion. The aims of this metanalysis were to evaluate the efficacy and safety of hAPC in the setting of Barret’s esophagus (BE) ablation and as an adjunct to colonic endoscopic mucosal resection (EMR). Four electronic databases were searched, and the results were analyzed by two independent authors. Random-effects meta-analyses of the proportions of endoscopic and histologic remission (for BE), recurrence, and post-procedure adverse events were performed using R. Studies’ reporting quality was also assessed. From the 979 identified records, 13 studies were included (10 regarding BE and three colonic EMR). The pooled percentages of endoscopic and histologic remission after hAPC for BE were 95% (95% confidence interval [CI] 91–99, I^2^ = 34) and 90% (95%CI 84–95, I^2^ = 46), respectively, while major adverse events and recurrence were registered in 2% (95%CI 0–5, I^2^ = 41) and 11% (95%CI 2–27, I^2^ = 11), respectively. Concerning hAPC-assisted EMR, the pooled percentages of major adverse events and recurrence were 5% (95%CI 2–10, I^2^ = 0) and 1% (95%CI 0–3, I^2^ = 40). Evidence suggests that the main advantages of hAPC are the increase in safety in the setting of BE ablation and the reduction of local recurrence after colonic EMR. Trials comparing hAPC with standard strategies are required to support its use for these indications.

## 1. Introduction

Advanced endoscopic techniques have significantly improved the morbidity and mortality associated with premalignant conditions (as Barrett’s esophagus) and premalignant lesions (as most large colonic polyps) [1].

While the evidence does not support prophylactic endoscopic therapy of non-neoplastic BE, patients with visible lesions associated to BE must undergo endoscopic resection in expert centers, followed by eradication of residual BE, while those harboring low- and high-dysplasia without visible lesions should be submitted to ablation [2]. Ideally, the ablative tool should allow complete and sustained endoscopic and histological eradication of all Barrett’s mucosa, be easy to apply, and be safe. Currently, the preferred method for eradication is radiofrequency ablation (RFA) [1,3], in which heat induces necrosis of the target mucosa. However, this technique has some important drawbacks, including the need for expensive material and frequent endoscope reintroduction, usually with an over-the-scope apparatus of challenging use [4]. Besides that, recurrence occurs in up to one-third of patients, while adverse events, particularly post-procedure pain, and strictures, are frequent [5]. Therefore, some alternatives have been proposed. Another option is argon plasma coagulation (APC) [6], a contact-free ablation technique using free-flow ionized argon gas. Notwithstanding, there are several factors influencing APC outcomes, such as the energy settings and the distance between the catheter and the mucosa, with an inconstant depth of tissue injury. A different modality is cryotherapy (cryogen-induced necrosis) [7], which has been considered comparable to RFA in terms of efficacy and may be used as salvage therapy.

Lately, the focus moved to hybrid argon plasma coagulation (hAPC). This technique combines conventional APC with the needleless high-velocity waterjet delivery of a lifting agent, allowing for submucosal expansion that reduces the risk of penetrating injury while avoiding extramural injection, making it possible to treat greater tissue depth than standard APC [8]. A recently published meta-analysis of seven retrospective non-controlled studies has suggested that hAPC is associated with high rates of complete remission of intestinal metaplasia than those reported for RFA, with a favorable safety profile [9]. More recently, additional studies have been published.

Another field where hAPC has been generating interest is in the adjuvant management of large (>20 mm) non-pedunculated colorectal lesions [10,11,12] which carry a significant risk of submucosal invasive cancer [13]. The mainstay for these polyps’ resection is endoscopic mucosal resection (EMR), mostly in a piecemeal fashion. However, post-EMR local recurrence occurs in up to 22% of the cases [14]. Therefore, adjuvant ablation of the post-EMR margins, using snare-tip soft coagulation (STSC) or APC, has been increasingly recommended to remove any microscopic tissue that could be left on resection margins, with a positive impact on recurrence rates (estimated as low as 5% in some reports) [15]. The use of hAPC for this setting is theoretically superior, as it also allows to safely ablate the polypectomy eschar bed, further eliminating residual neoplastic tissue [16].

This study aims to systematically review the literature and to perform a meta-analysis of the efficacy and safety of hAPC in the setting of BE and as an adjunct to colonic EMR. We decided to explore both indications to provide a complete overview of all evidence available on this novel technique, rendering guidance to future studies and upcoming clinical practice.

## 2. Materials and Methods

### 2.1. Search Strategy

This study followed the Cochrane collaboration guidelines for systematic reviews [17] and the Preferred Reporting Items for Systematic Reviews and Meta-Analyses (PRISMA) [18].

The online search included MEDLINE (via PubMed), Web of Science, Scopus, and Cochrane Central Register of Controlled Trials (CENTRAL). Databases were searched from inception up to 15 December 2022, using the terms “(hybrid argon plasma coagulation OR argon plasma coagulation) AND (colon OR endoscopic mucosal resection OR Barrett’s esophagus)”. This query was used for PubMed search and adjusted for the other databases (Supplementary Table S1). The reference lists of the included manuscripts were hand-searched to identify further relevant publications.

### 2.2. Inclusion and Exclusion Criteria

The inclusion criteria were: [i] studies evaluating the use of hAPC in BE (alone or in combination with resection techniques) or as an adjunct to endoscopic mucosal resection (EMR) of colonic polyps. Both single-arm and double-arm (comparing hybrid APC with other strategies) studies were included. No language or publication date restrictions were imposed. The exclusion criteria were: [i] systematic or narrative reviews; [ii] guidelines, expert opinions, and editorials; and [iii] animal studies. No language or publication date restrictions were imposed. Whenever there were multiple reports of the same study, those with the most complete data were included.

### 2.3. Study Selection and Data Collection

Two authors (MME and RP) independently screened the literature. First, the titles and abstracts of the identified studies were carefully analyzed, and those that did not meet the eligibility criteria were excluded. The full texts of the remaining studies were evaluated to determine their inclusion or exclusion. The studies selected by each author were compared and disagreements were solved by discussion. The following information was collected from each study: study design, population characteristics, prior treatments, hybrid APC details, comparator (in double-arm studies), endoscopic surveillance, number of patients, follow-up duration, proportions of patients achieving endoscopic and histologic remission (for BE), presenting recurrence, and post-procedure adverse events. Differences in data extraction were settled by consensus.

### 2.4. Quality Assessment

Studies’ methodology and reporting quality was assessed independently by two authors using the Critical Appraisal Skills Programme (CASP) checklist for cohort studies (25). This validated tool assesses the relevance, validity, and results of each study using 12 categories. Each category was rated using a color scheme: green, when all parameters of a given item were met; yellow, if parameters were met partially or the information was incomplete; red, if not met. Funnel plots’ asymmetry and Egger’s test were used to estimate the risk of publication bias.

### 2.5. Statistical Analysis

The endpoints of this meta-analysis were: [i] proportions of endoscopic and histologic remissions after using hAPC for BE ablation (defined as the macroscopic impression of complete BE eradication and absence of intestinal metaplasia on biopsies; respectively); [ii] proportion of post-procedure adverse events after applying hAPC to either BE or colonic lesions; and [iii] proportion of patients presenting BE recurrence (reappearance of endoscopic changes suggesting BE and/or detection of intestinal metaplasia on biopsies) or post-EMR recurrence (presence of dysplastic tissue on follow-up endoscopy). Data were extracted from each individual study. The recurrence rate adjusted for follow-up was estimated, for each study, by dividing the number of recurrences (denominator) by the product of the number of patients * follow-up duration (numerator).

The R software, version 4.1.0 (R Foundation for Statistical Computing, Vienna, Austria), was used to analyze data and to generate the funnel and forest plots. Statistical heterogeneity was assessed using Cochran’s Q test and I^2^ statistic; an I^2^ below 25% suggested low heterogeneity, while values between 25–50% and above 50% corresponded to moderate and high heterogeneity, respectively. Pooled proportional meta-analyses were done using random-effects (restricted maximum likelihood [REML]) and fixed-effects models, using the ‘metaprop’ function from the ‘meta’ package of the R statistical programming [19]. Considering the small number of events, arcsine-transformed proportions were used [20]. The pooled estimates were then back-transformed, and the pooled results were reported as proportions. In addition, a sensitivity analysis was performed to assess the influence of any individual study on the overall results. A *p*-value lower than 0.05 was considered statistically significant.

## 3. Results

### 3.1. Literature Search and Study Selection

**Figure 1:**
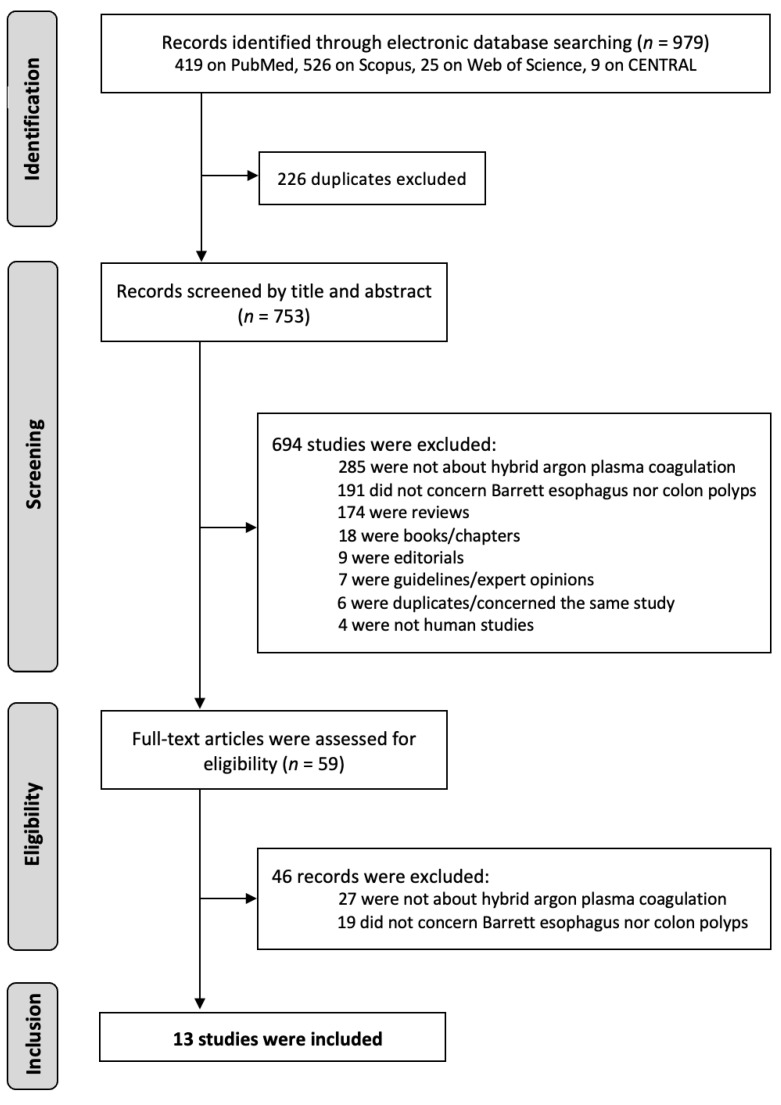
Flow diagram of studies selection and data collection process.

The search yielded 979 records: 419 were found in PubMed, 526 in Scopus, 25 in Web of Science, and nine in CENTRAL. Following the removal of duplicates, 753 records remained, of which 694 were excluded. Then, 59 studies were assessed for eligibility, and 13 were included (Figure 1): 10 evaluated hAPC for BE ablation, while three concerned hAPC-assisted EMR.

### 3.2. Characteristics of Included Studies

#### 3.2.1. Studies on Barrett’s Esophagus

The characteristics of the studies evaluating the use of hAPC for BE are summarized in Table 1. Of the ten studies included, half were prospective [4,8,21,22,23], and only two compared hAPC with other therapeutic modalities (EMR [24] and radiofrequency ablation [25]). Globally, 318 patients were submitted to hAPC; however, the sample size varied widely. Around 84.0% of patients had already received prior treatments for BE (RFA, EMR or cryotherapy). The hAPC settings were similar among the studies, consisting of submucosal injection of normal saline using Erbejet, followed by BE ablation with 60–70 watts using a contact-free thermal hAPC probe (first passage) and then 40–50 watts (for remaining islets or EMR defect, after using a transparent cap to scrape off the coagulated remainders). Mean hAPC session duration varied from 3.5 ± 1.4 min [4] to 26 min (range 5–105) [21]. Acid suppressive therapy was always introduced after ablation, and hAPC sessions were continued at variable periods (every three to twelve weeks) until complete BE eradication or up to a defined number of sessions (mostly five), after which the case was considered a failure. The follow-up duration was variable, ranging from three [8] to 24 [4,21] months. Post-procedure surveillance started mostly at three months and was repeated periodically. Biopsies were mostly done according to the Seattle protocol.

#### 3.2.2. Studies on Colonic Endoscopic Mucosal Resection

Three studies [10,11,12] evaluated the use of hAPC after EMR of colonic polyps, two of which were prospective [10,11] (Table 2). These reports enrolled 164 subjects, from whom 195 non-pedunculated polyps were removed (166 with hAPC-assisted EMR). Polyps’ size was similar among studies, as was the rate of complete lifting; however, the percentage of *en bloc* resection varied: 6% in the study by Motchum et al. [10] and 28.8% in that by Levenick et al. [12]. Regarding the hAPC technique, even though the settings were similar (effect 30–50, prior to thermal ablation with 40 watts), both the eschar base and peripheral edges were always ablated only in two of the studies [11,12]. The surveillance strategy and follow-up duration (six months) were similar, and all reported the rate of recurrence and post-procedure adverse events.

### 3.3. Hybrid Argon Plasma Coagulation for BE

#### 3.3.1. Endoscopic and Histologic Remission

**Figure 2:**
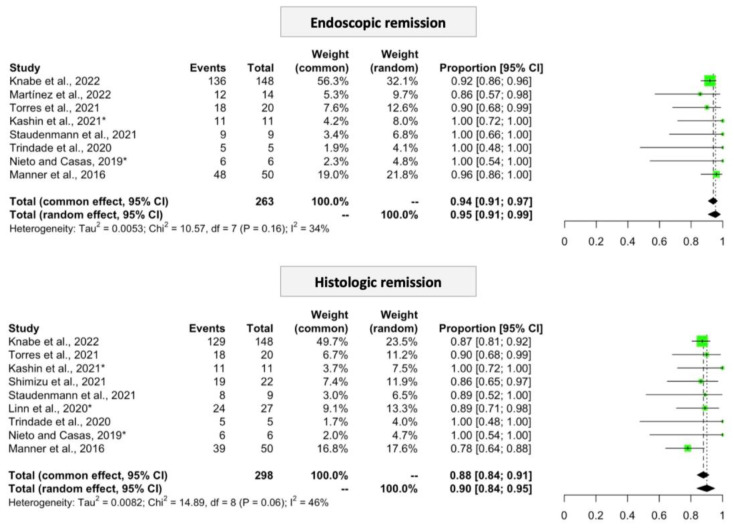
Proportions of achievement of endoscopic and histologic remission after hybrid argon plasma coagulation (hAPC) for Barrett’s esophagus (BE) ablation. Events = number of patients achieving endoscopic or histologic remission; total = number of patients evaluated in which study. *Study available only in the abstract form. References of the studies: Knabe et al., 2022 [21]; Martínez et al., 2022 [24]; Torres et al., 2021 [26]; Kashin et al., 2021 [22]; Staudenmann et al., 2021 [4]; Trindade et al., 2020 [27]; Nieto and Casas, 2019 [28]; Manner et al., 2016 [8]; Linn et al., 2020 [25]; Shimizu et al., 2021 [23].

The proportion of patients achieving endoscopic remission, corresponding to the macroscopic absence of BE, was evaluated in eight studies and ranged between 0.86 [24] and 1.00 [4,22,27,28] (Table 1). The pooled proportion of endoscopic remission was 0.95 (95% confidence interval [CI] 0.91–0.99); I^2^ = 34%, *p* = 0.16) (Figure 2).

Histologic remission (complete eradication of intestinal metaplasia) was assessed in all studies apart from one [24] and varied among 0.78 [8] (patients with residual non-neoplastic BE after EMR) and 1.00 [22,27,28] (patients with neoplastic BE), being the pooled histologic remission 0.90 (95%CI 0.84–0.95; I^2^ = 46%, *p* = 0.06). The histologic remission of the group submitted to hAPC was 14.1% [25] higher than that obtained after RFA, in the study carried out by Linn et al.; however, this difference was not statistically different.

#### 3.3.2. Procedure-Related Adverse Events

**Figure 3:**
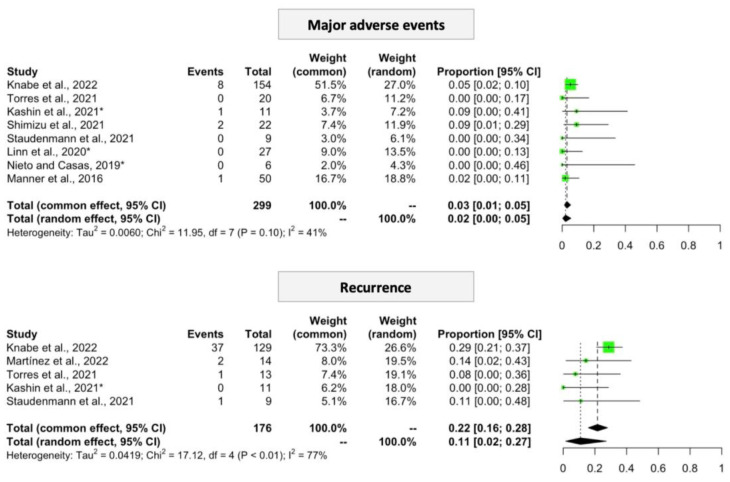
Proportions of adverse events and recurrence after hybrid argon plasma coagulation (hAPC) for Barrett’s esophagus (BE) ablation. Events = number of patients presenting major adverse events or recurrence; total = number of patients evaluated in which study. *Study available only in the abstract form. References of the studies: Knabe et al., 2022 [21]; Torres et al., 2021 [26]; Kashin et al., 2021 [22]; Shimizu et al., 2021 [23]; Staudenmann et al., 2021 [4]; Linn et al., 2020 [25]; Nieto and Casas, 2019 [28]; Manner et al., 2016 [8]; Martínez et al., 2022 [24].

The occurrence of major procedure-related adverse events was reported in eight studies [4,8,21,22,23,25,26,28] and ranged from 0% (in four studies [4,25,26,28]) to 9% (in two [22,23]; Table 1), with a pooled proportion of 0.02 (95%CI 0.00–0.05; I^2^ = 41%, *p* = 0.10) (Figure 3). Only one episode of perforation and one of major bleeding were reported [21]; both complications were amenable to endoscopic therapy. The ten strictures registered [8,21,22,23] were managed with endoscopic dilatation. The rate of minor events (transient chest discomfort, heartburn, or odynophagia) ranged from 11.1% [4] to 20.1% [21].

#### 3.3.3. Recurrence

Five studies evaluated the recurrence of intestinal metaplasia [4,9,10,13,15]. Knabe et al. [9] found a histologic recurrence rate of 29.2% (Table 1; Figure 3) after 24 months of follow-up. Importantly, 22 of these cases (59.5%) were only detected by biopsy as no macroscopic signs of BE had been detected. Additionally, about one-third (*n* = 11) of the recurrences were only detected 23 months after the first hAPC treatment. The recurrence rates reported by the other studies were lower: 0.0% (4.5 months follow-up [15]), 7.7% (6 months), 11.1% (24 months), and 14.3% (12 months). The pooled proportion of recurrence was 0.11 (95%CI 0.02–0.27, I^2^ = 77%, *p* < 0.01).

### 3.4. Hybrid Argon Plasma Coagulation after Colonic EMR

#### 3.4.1. Procedure-Related Adverse Events

**Figure 4:**
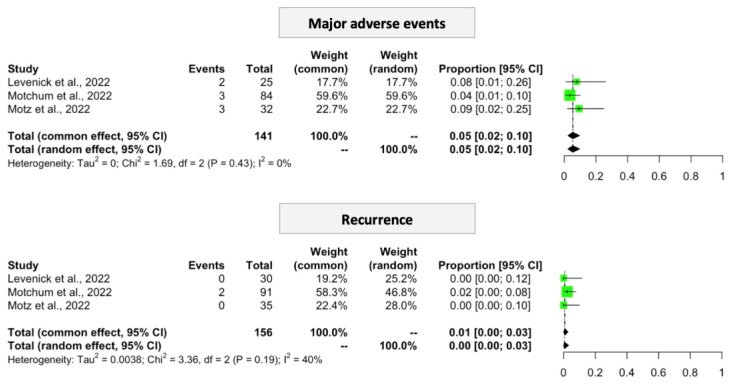
Proportions of major adverse events and of recurrence at 6 months after hybrid argon plasma coagulation (hAPC)-assisted endoscopic mucosal resection (EMR) of colonic lesions with more than 20 mm. Events = number of patients presenting major adverse events or recurrence; total = number of patients evaluated in which study. References of the studies: Levenick et al., 2022 [12]; Motchum et al., 2022 [10]; Motz et al., 2022 [11].

Major post-procedure adverse events were registered in eight out of 141 hAPC-treated patients, with a pooled proportion of 0.05 (95%CI 0.02–0.10, I^2^ = 0%, *p* = 0.43) (Table 2, Figure 4). All apart from one corresponded to major bleeding, defined as that requiring hospitalization, blood transfusion, and/or intervention for hemostasis and occurring up to 30 days after EMR. The single comparative study [12] showed a tendency towards higher safety of hAPC in comparison to standard EMR (major adverse events in 8.0 and 17.4%, respectively). The rate of intraprocedural bleeding requiring intervention was reported only once (13.1%) [10].

#### 3.4.2. Recurrence

Only one study identified local recurrence in the six-month follow-up, in two out of 91 patients submitted to hAPC-assisted EMR [10], corresponding to a pooled proportion of 0.01 (95%CI 0.00–0.03; I^2^ = 40%, *p* = 0.19) (Figure 4).

### 3.5. Publication Bias and Reporting Quality

Funnel plots’ analysis suggested a low risk of publication bias (Supplementary Figures S1 and S2). Likewise, the results of Egger’s test on the different proportions were not significant (*p*-values between 0.116 and 0.389). Concerning reporting quality (Supplementary Figure S3), all studies clearly stated the issue under analysis and provided believable results that were deemed to have practical implications. However, most failed to use designs that minimized bias and the effect of confounding factors, while some had short follow-up duration. Additionally, three studies were only available as conference abstracts, reducing data availability.

## 4. Discussion

The last decades have been bright for gastrointestinal endoscopy. More than ever, the focus is to improve detection, while maximizing the efficiency and safety of therapeutic modalities. To the best of our knowledge, this is the first study systematically evaluating the efficacy and safety of hAPC, a recent and promising technique that combines submucosal injection and standard APC in a single probe, in the settings of BE and colonic EMR.

This study included 13 studies, ten concerning the application of hAPC for BE ablation. Due to a comprehensive search, our study was able to include more studies than those polled on a recently published meta-analysis regarding only APC for BE [9]. In this setting, the pooled endoscopic (no macroscopic signs of BE) and histologic (complete eradication of intestinal metaplasia) remissions were 95% (95%CI 91–99) and 90% (95%CI 84–95), respectively, being the heterogeneity among studies moderate. On the other hand, standard “non-lifting” APC had been associated with lower percentages of BE eradication, ranging from 38% to 77% depending presumably on the voltage used and on the depth of tissue damage as well as on follow-up duration [8]. The apparently higher efficacy of hAPC may be understood considering that the submucosal cushion allows ablating larger and deeper BE areas [4], yet no head-to-head studies have systematically evaluated this question. Regarding other alternatives, histologic remission after RFA was estimated to be 78% (95%CI 70–86), in a metanalysis of 18 studies [29], while the primary use of cryotherapy eliminated intestinal metaplasia in 64% (95%CI 53–75, pooled estimate of 13 reports [30]). To date, a single study with low reporting quality compared hAPC with the mainstay RFA [25], showing a tendency towards the superiority of hAPC. In our metanalysis, the pooled recurrence after hAPC was 11% (95%CI 2–27). Considering that the included studies were heterogenous, particularly regarding follow-up duration, we also calculated the recurrence rate adjusted for time (13.7 per 100 patient-years of follow-up). These values are in line with the reported for other techniques: 9.5–13% and 9.6 per 100 patient years for RFA [31,32] and 12.7% and 19.1 per 100 patient years for cryotherapy [33,34].

Concerning side effects, the pooled percentage of major adverse events following hAPC for BE was 2% (95%CI 0–5), corresponding almost exclusively to strictures amenable to endoscopic dilation (10 cases in 318 patients). Minor events, including chest pain and dysphagia, have been reported to occur in 11–20% [4,21]. These percentages are considerably lower than those reported for the other BE ablation techniques, allowing us to hypothesize that safety may be the hallmark of hAPC. In fact, RFA has a significant symptoms burden, being associated with chest pain that lasts for an average of 14 days in up to 95% of the patients (major pain in two-thirds) and with transient dysphagia in more than 50%, while major adverse events like strictures, bleeding, and perforation are estimated to occur in 6%, 1%, and 0.6%, respectively [5]. Likewise, the use of standard APC for BE has been associated with chest pain in 47–74%, dysphagia in 5–15%, stenosis in 0–3%, and perforations in 0–1% of the patients [35]. Besides this, the risk of malignant transformation of the glands buried with standard APC cannot be neglected [4]. Despite being associated with less damage to tissue architecture, cryotherapy is still associated with a notable rate of side effects (around 12%), mostly strictures and chest pain [30]. The best safety profile of hAPC is associated with the submucosal needle-free expansion, which reduces the coagulation depth to half of that observed for standard APC, protecting the integrity of the muscularis propria layer [8].

Regarding the use of hAPC as an adjunct to EMR of large colonic polyps, three studies enrolling 164 patients were available. The pooled recurrence at six months was 1% (95%CI 0–3), which was lower than those previously pooled for both APC (9%, 95%CI 4–19) and STSC (4%, 95%CI 2–8) [10]. The higher efficacy of hAPC-assisted EMR may be due to the ablation of both macroscopically normal eschar margins and base (the latter not amenable to ablation with APC or STSC due to safety issues), as microscopic neoplastic tissue is probably present in both locations. Interestingly, the single study detecting recurrences was the one where the eschar surface was not systematically ablated (complete only in 20% of the cases), which strengthens this hypothesis.

The pooled percentage of adverse events occurring 30 days after hAPC-assisted EMR was 5% (95%CI 2–10), corresponding mostly to major bleeding. Intraprocedural bleeding occurred in 13% of patients included in the single study that quantified this parameter [10]. These values are identical to those reported for STSC-assisted EMR, where intraprocedural and delayed bleeding are estimated to occur in 10% and 6.5%, respectively [13]. However, no studies compared directly hAPC with the currently preconized margins’ ablation methods (standard APC and STSC).

Even though the ability to extrapolate findings remains limited, the results of this metanalysis suggest that the main advantage of hAPC for BE is an increase in safety, while for colonic EMR is the reduction of local recurrence. This study has some strengths: (i) it is the first systematic review evaluating the efficacy and safety of hAPC; (ii) the search strategy was exhaustive to maximize the likelihood of identifying all relevant studies; (iii) a detailed quality assessment was performed; and (iv) a random effects model was used to provide more conservative estimates. Notwithstanding, it also has limitations. First, evidence comes mostly from retrospective studies, with modest sample sizes. Second, the number of studies was limited, restraining internal and external validity. Indeed, despite the efforts to identify all relevant studies, hAPC is a very recent technique, being the body of evidence inherently small. Third, the assessment of recurrence after hAPC may be biased, as the procedures were done in tertiary high-volume centers; follow-up durations were overall short; and recurrence was evaluated by endoscopists aware of the allocated treatment (this is particularly relevant for colonic EMR, as no scar biopsies were taken).

In conclusion, the evidence indicates that hAPC offers significant benefits over traditional techniques, including enhanced safety during BE ablation and a decrease in local recurrence following colonic EMR. Further high-quality research, including randomized trials, is needed to clarify the open questions, particularly to compare hAPC versus RFA for BE and STSC versus hAPC for colonic EMR. Finally, it will be important to formally evaluate the cost-effectiveness of hAPC for the two indications, particularly for EMR where the almost null additional direct costs of the STSC strategy may be tough to beat [36]. Last but not the least, it reinforces the need to keep patients with premalignant conditions under protocoled surveillance, especially for BE, as the technical developments have not yet been able to totally control the natural disease course.

## Figures and Tables

**Table 1:** Summary of the studies evaluating hybrid argon plasma coagulation (hAPC) for Barrett esophagus (BE) ablation. Barrett esophagus (BE); endoscopic mucosal resection (EMR); hybrid argon plasma coagulation (hAPC); not reported (NR); radiofrequency ablation (RFA); watt (W).

Study	Study Type	Population	Prior Treatment Modalities	Hybrid APC Details	Surveillance	N	Follow-Up	Endoscopic Remission	Histologic Remission	Recurrence	Procedure-Related Complications
Knabe et al., 2022 [21]	Prospective, multicentric, single arm	Patients with macroscopically invisible neoplastic BE	EMR (89.6%), treatment-naïve (10.4%)	Submucosal injection of sodium chloride 0.9% with ERBEjet 2 (ERBE, Germany), prior to ablation (first 60–70 W; then 40 W for the remaining islets); mean 2.7 sessions (range 1–5)	Endoscopy with 4-quadrant biopsies from former BE, neo-Z line and neosquamous epithelium (every 1–2 cm) at 3, 6, 12 and 24 months	154 (80.9% male; mean age 64.2 [range 42–84])	24 months	136/148 = 92.6%	129/148 = 87.2%	37/129 = 29.2%	1/154 = 0.6% (perforation), 6/154 = 3.9% (stricture), 1/154 = 0.6% (bleeding); 31/154 = 20.1% (minor events)
Martínez et al., 2022 [24]	Retrospective cohort study, unicentric, double arm	Patients with biopsy-proven BE + low-grade flat dysplasia	Treatment-naïve patients	Submucosal injection with ERBEjet2 prior to ablation (60 W, effect 2 in the first section; 50 W, effect 2 in subsequent sections)	Endoscopy at 6 and 12 months	29 (58.6% male, mean age 50.5 [range 27–81]	12 months	12/14 = 85.7% versus 12/15 = 80.0% (EMR)	NR	2/14 = 14.3% versus 3/15 = 20.0% (EMR)	NR
Torres et al., 2021 [26]	Retrospective, cohort study, unicentric, single arm	Patients with biopsy-proven BE + low-grade flat dysplasia	NR	Hybrid-APC in the areas of dysplasia (2 quadrants per session); 60 W and effect 2 (first session), 50 W and effect 2 (subsequent); mean 1.5 sessions (range 1–4)	Endoscopy with biopsies from former BE at 3 and 6 months	20 (55.0% male, mean age 50.5 [range 27–81])	6 months	18/20 = 90.0%	18/20 = 90.0%	1/13 = 7.7%	0/20 = 0.0%
Kashin et al., 2021 (abstract) [22]	Prospective, unicentric, single arm	Patients with biopsy-proven BE + flat low-grade dysplasia	Treatment-naïve (54.5%), EMR (45.5%)	Submucosal injection with ERBEjet2 prior to ablation (first with 60 W, effect 2; remaining islets treated with 40 W, effect 2); mean of 1.6 sessions (range 1–3)	Endoscopy with 4-quadrant biopsies from former BE at 3, 6 months and then annually	11 (45.5% male, mean age 46 [range 25–63])	Median 4.5 months	11/11 = 100.0%	11/11 = 100.0%	0/11 = 0.0%	1/11 = 9.0% (stricture)
Shimizu et al., 2021 [23]	Prospective, unicentric, single arm	Patients with residual BE (54.4% with flat neoplasia)	Treatment-naïve (36.0%), EMR (22.7%), RFA (50.0%), and cryotherapy (13.6%)	Submucosal injection of 0.9% methylene blue solution with ERBEjet2, 40–50 W, effect 2, prior to ablation (first with 60 W, effect 2; remaining islets treated with 40 W, effect 2); up to 3 sessions (range 2–4)	Endoscopy with biopsies at the neo Z-line and biopsies in at least one level in the area of the former BE at 3 months	22 (81.8% male, mean age 67.8 [range 49–83])	Average 134.7 days	NR	19/22 = 86.4%	NR	2/22 = 9.1% (strictures)
Staudenmann et al., 2021 [4]	Prospective, unicentric, single arm	Patients with biopsy-proven BE, with flat low- or high-grade dysplasia, or T1a adenocarcinoma	Treatment-naïve (72.8%), RFA (9.1%) or EMR (18.2%)	Submucosal injection of 0.9% sodium chloride solution with ERBEjet2, effect 50, prior to ablation (60–70 W, effect 2)	Endoscopy with biopsies at the neo Z-line and biopsies in at least one level in the area of the former BE at 3, 6, 9, 12, 18, 24 months	9 (72.7% male, mean age 68.2 ± 8.0 years-old)	24 months	9/9 = 100.0%	8/9 = 88.9%	1/9 = 11.1%	0/9 = 0.0%;1/9 = 11.1% (minor event)
Linn et al., 2020 (abstract) [25]	Retrospective cohort study, unicentric, double arm	Patients with residual BE	NR	NR	Endoscopy with 4-quadrant biopsies from former BE at 3 and 6 months	54 (83.0% male, mean age 66.5 years-old)	6 months	NR	24/27 = 88.9% versus 20/27 = 74.1%	NR	0/27 = 0.0% versus 12/27 = 44.4% (for RFA, 4 strictures)
Trindade et al., 2020 [27]	Retrospective, case reports, unicentric	Refractory residual BE (in 2 cases with non-visible neoplasia)	RFA, 60.0% also refractory to cryotherapy	Submucosal injection with ERBEjet, effect 50, prior to ablation (40 W in the EMR defect, 60 W in the remaining nondysplastic mucosa); mean 2.2 sessions (range 2–3)	Endoscopy 3 and 6 months later; no further details	5 (60.0% male; mean age 66.8 [range 51–76])	Not defined	5/5 = 100.0%	5/5 = 100.0%	NR	NR
Nieto and Casas, 2019 (abstract) [28]	Retrospective cohort study, unicentric, single arm	BE with persistent flat dysplasia	Failed RFA and cryotherapy	Hybrid APC (no further details), mean 2.3 sessions	Endoscopy 6 months later; no further details	6 (84.0% male, mean age 63)	6 months	6/6 = 100%	6/6 = 100%	NR	0/6 = 0.0%
Manner et al., 2016 [8]	Prospective, unicentric, single arm	Patients with residual non-neoplastic BE	EMR (100%)	Submucosal injection of sodium chloride 0.9% with ERBEjet, prior to ablation (first with 50–60 W, effect 2; then 40 W for the remaining islets); median sessions 3.5 (range 1–10)	Endoscopy with 4 quadrant biopsies from former BE, neo-Z line and neosquamous epithelium at 3 months (every 2 cm)	50 (92.0% male; mean age 62.0 [range 42–79])	3 months	48/50 = 96.0% (PP)	39/50 = 78.0%	NR	1/50 = 2% (stricture), 11/50 = 22% (minor events)

**Table 2:** Summary of the studies evaluating hybrid argon plasma coagulation (hAPC)-assisted endoscopic mucosal resection (EMR) of colonic lesions. Endoscopic mucosal resection (EMR); hybrid argon plasma coagulation (hAPC); not reported (NR); radiofrequency ablation (RFA); standard EMR (sEMR); watt (W).

Study	Study Type	Population	Hybrid APC Details	Comparator	Surveillance	N	Follow-Up	Polyps’ Characteristics	Recurrence	Procedure-Related Complications
Levenick et al., 2022 [12]	Retrospective cohort study, unicentric, double arm	Patients who underwent EMR for non-pedunculated colonic polyps > 20 mm	Submucosal injection (normal saline and contrast agent) with ERBEjet effect 30–50, prior to thermal ablation (flow of 0.8 L/min, 40 W; done on both eschar base and peripheral edges)	Standard EMR (*n* = 29 polyps)	Surveillance colonoscopy at 6-months	48 (54.2% male, mean age 66.1); 59 polyps removed (30 with APC-assisted EMR)	6 months	Mean size 31.6 mm (SD 13.7), 66.1% in the right colon; en-bloc resection in 28.8%; lift adequacy differed (non-lifting in 10.3% of the sEMR group versus 3.3% of the hAPC)	0/30 = 0.0% versus 6/29 = 20.7% (standard EMR)	2/25 = 8.0% versus 4/23 = 17.4% for sEMR (bleeding)
Motchum et al., 2022 [10]	Prospective, multicentric, single arm	Patients who underwent EMR for non-pedunculated colonic polyps > 20 mm	Submucosal injection with ERBEjet, effect 30–50, prior to ablation (flow of 0.8 L/min, 40 W); the ablation of the peripheral edges was done in all patients; eschar surface was only ablated in 78% (complete in 20%, partial in 58%)	No	Surveillance colonoscopy at 4–6 months	84 (53.6% male, median age 66.3 [range 18–89]); 101 polyps removed	6 months	Median polyp size 30.9 mm (range 20–60 mm), 83.0% in the right colon, en-bloc resection in 6.0%; non-lifting in 8.0%; prophylactic clipping in 13.1%	2.2% (2/91)	2/84 = 2.4% (bleeding); 1/84 = 1.2% (microperforation)
Motz et al., 2022 [11]	Prospective, unicentric, single arm	Patients who underwent EMR for non-pedunculated colonic polyps > 20 mm	Submucosal injection (sodium chloride [0.9%] ± hetastarch), prior to thermal ablation (flow of 0.8 L/min, 40 W; done on both eschar base and peripheral edges)	No	Surveillance colonoscopy at 6-months	32 (62.5% male, mean age 64.6 [range 50–78]); 35 polyps removed	6 months	Median polyp size 27.0 mm (IQR 14.5); 65.9% in the right colon; non-lifting in 4.6%; prophylactic clipping in 82.5%	0/35 = 0.0%	3/32 = 7.5% (bleeding)

## Data Availability

The data underlying this article will be shared on reasonable request to the corresponding author.

## Supplementary Materials

The following supporting information can be downloaded at: https://www.mdpi.com/article/10.3390/biomedicines11041139/s1, Supplementary Table S1: Search strategy. Supplementary Figure S1: Funnel plots for the proportions of outcomes’ achievement after hybrid argon plasma coagulation (hAPC) for Barrett esophagus (BE) ablation. Supplementary Figure S2: Funnel plots for the proportions of outcomes’ achievement after hybrid argon plasma coagulation (hAPC)-assisted endoscopic mucosal resection (EMR) of colonic lesions with more than 20 mm. Supplementary Figure S3: Results of the reporting quality analysis, using the Critical Appraisal Skills Programme (CASP) checklist.

## References

[1] Nieuwenhuis E.A., van Munster S.N., Curvers W.L., Weusten B.L.A.M., Alvarez Herrero L., Bogte A., Alkhalaf A., Schenk B.E., Koch A.D., Spaander M.C.W. (2022). Impact of Expert Center Endoscopic Assessment of Confirmed Low Grade Dysplasia in Barrett’s Esophagus Diagnosed in Community Hospitals. Endoscopy.

[2] Weusten B., Bisschops R., Coron E., Dinis-Ribeiro M., Dumonceau J.-M., Esteban J.-M., Hassan C., Pech O., Repici A., Bergman J. (2017). Endoscopic Management of Barrett’s Esophagus: European Society of Gastrointestinal Endoscopy (ESGE) Position Statement. Endoscopy.

[3] van Munster S.N., Pouw R.E., Sharma V.K., Meijer S.L., Weusten B.L.A.M., Bergman J.J.G.H.M. (2021). Radiofrequency Vapor Ablation for Barrett’s Esophagus: Feasibility, Safety and Proof of Concept in a Stepwise Study with in Vitro, Animal, and the First in-Human Application. Endoscopy.

[4] Staudenmann D.A., Skacel E.P., Tsoutsman T., Kaffes A.J., Saxena P. (2021). Safety and Long-Term Efficacy of Hybrid-Argon Plasma Coagulation for the Treatment of Barrett’s Esophagus: An Australian Pilot Study. Int. J. Gastrointest. Interv..

[5] Overwater A., Elias S.G., Schoon E.J., Bergman J.J.G.H.M., Pouw R.E., Weusten B.L.A.M. (2022). The Course of Pain and Dysphagia after Radiofrequency Ablation for Barrett’s Esophagus-Related Neoplasia. Endoscopy.

[6] Shaheen N.J., Sharma P., Overholt B.F., Wolfsen H.C., Sampliner R.E., Wang K.K., Galanko J.A., Bronner M.P., Goldblum J.R., Bennett A.E. (2009). Radiofrequency Ablation in Barrett’s Esophagus with Dysplasia. N. Engl. J. Med..

[7] Fasullo M., Shah T., Patel M., Mutha P., Zfass A., Lippman R., Smallfield G. (2022). Outcomes of Radiofrequency Ablation Compared to Liquid Nitrogen Spray Cryotherapy for the Eradication of Dysplasia in Barrett’s Esophagus. Dig. Dis. Sci..

[8] Manner H., May A., Kouti I., Pech O., Vieth M., Ell C. (2016). Efficacy and Safety of Hybrid-APC for the Ablation of Barrett’s Esophagus. Surg. Endosc..

[9] Shah S.N., Chehade N.E.H., Tavangar A., Choi A., Monachese M., Chang K.J., Samarasena J.B. (2023). Hybrid Argon Plasma Coagulation in Barrett’s Esophagus: A Systematic Review and Meta-Analysis. Clin. Endosc..

[10] Motchum L., Levenick J.M., Djinbachian R., Moyer M.T., Bouchard S., Taghiakbari M., Repici A., Deslandres É., von Renteln D. (2022). EMR Combined with Hybrid Argon Plasma Coagulation to Prevent Recurrence of Large Nonpedunculated Colorectal Polyps (with Videos). Gastrointest. Endosc..

[11] Motz V.L., Lester C., Moyer M.T., Maranki J.L., Levenick J.M. (2022). Hybrid Argon Plasma Coagulation-Assisted Endoscopic Mucosal Resection for Large Sessile Colon Polyps to Reduce Local Recurrence: A Prospective Pilot Study. Endoscopy.

[12] Levenick J.M., Groff A.J., Manzo C., Lester C., Maranki J.L. (2022). Hybrid APC Colon EMR, A Novel Approach to Reduce Local Recurrence. Tech. Innov. Gastrointest. Endosc..

[13] Chandan S., Facciorusso A., Ramai D., Deliwala S., Mohan B.P., Kassab L.L., Draganov P.V., Othman M.O., Kochhar G.S. (2022). Snare Tip Soft Coagulation (STSC) after Endoscopic Mucosal Resection (EMR) of Large (>20 Mm) Non Pedunculated Colorectal Polyps: A Systematic Review and Meta-Analysis. Endosc. Int. Open.

[14] Kemper G., Turan A.S., Schoon E.J., Schrauwen R.W.M., Epping L.S.M., Gerges C., Beyna T., Neuhaus H., Gündug U., Siersema P.D. (2021). Endoscopic Techniques to Reduce Recurrence Rates after Colorectal EMR: Systematic Review and Meta-Analysis. Surg. Endosc..

[15] Kaltenbach T., Anderson J.C., Burke C.A., Dominitz J.A., Gupta S., Lieberman D., Robertson D.J., Shaukat A., Syngal S., Rex D.K. (2020). Endoscopic Removal of Colorectal Lesions-Recommendations by the US Multi-Society Task Force on Colorectal Cancer. Gastrointest. Endosc..

[16] Manner H., Neugebauer A., Scharpf M., Braun K., May A., Ell C., Fend F., Enderle M.D. (2014). The Tissue Effect of Argon-Plasma Coagulation with Prior Submucosal Injection (Hybrid-APC) versus Standard APC: A Randomized Ex-Vivo Study. United Eur. Gastroenterol. J..

[17] Higgins J., Green S., The Cochrane Collaboration (2011). Cochrane Handbook for Systematic Reviews of Interventions Version 5.1.0.

[18] Moher D., Liberati A., Tetzlaff J., Altman D. (2010). Preferred Reporting Items for Systematic Reviews and Meta-Analyses: The PRISMA Statement—Preferred Reporting Items for Systematic Reviews and Meta-Analyses. Int. J. Surg..

[19] Balduzzi S., Rücker G., Schwarzer G. (2019). How to Perform a Meta-Analysis with R: A Practical Tutorial. Evid. Based. Ment. Health.

[20] Elli L., Casazza G., Locatelli M., Branchi F., Ferretti F., Conte D., Fraquelli M. (2017). Use of Enteroscopy for the Detection of Malignant and Premalignant Lesions of the Small Bowel in Complicated Celiac Disease: A Meta-Analysis. Gastrointest. Endosc..

[21] Knabe M., Beyna T., Rösch T., Bergman J., Manner H., May A., Schachschal G., Neuhaus H., Kandler J., Weusten B. (2022). Hybrid APC in Combination With Resection for the Endoscopic Treatment of Neoplastic Barrett’s Esophagus: A Prospective, Multicenter Study. Am. J. Gastroenterol..

[22] Kashin S.V., Kuvaev R., Nadezhin A.S., Kraynova E.A., Nekhaykova N. (2016). The New Hybrid Argon Plasma Coagulation (Hybrid APC) for Endoscopic Ablation of Barrett’s Esophagus (BE): The Results of the Pilot Trial. Gastrointest. Endosc..

[23] Shimizu T., Samarasena J.B., Fortinsky K.J., Hashimoto R., El Hage Chehade N., Chin M.A., Moosvi Z., Chang K.J. (2021). Benefit, Tolerance, and Safety of Hybrid Argon Plasma Coagulation for Treatment of Barrett’s Esophagus: US Pilot Study. Endosc. Int. Open.

[24] Martínez I.M., Quintanilla R.A.B., Torres M.C.A., Morera N.D. (2022). Endoscopic Treatment of Barrett’s Esophagus with Low- and High-Grade Dysplasia. Med. Electrónica.

[25] Linn B., Mangels-Dick T., Clemens M.A., Hanada Y., Roy B., Genere J.R., Wang K.K. (2020). Hybrid Argon Plasma Coagulation and Radiofrequency Ablation in Barrett’s Esophagus. Gastrointest. Endosc..

[26] Torres M.C., Quintanilla R.A.B., Megías E.M.d.O., Escobar V.M.A., Contino N.C.A., Menocal J.L.G., Jiménez F.N.P. (2021). Hybrid-APC Therapeutic Response in Patients with Low-Grade Dysplasia in Barrett’s Esophagus. Arch. Cuba. Gastroenterol..

[27] Trindade A.J., Wee D., Wander P., Stewart M., Lee C., Benias P.C., McKinley M.J. (2020). Successful Treatment of Refractory Barrett’s Neoplasia with Hybrid Argon Plasma Coagulation: A Case Series. Endoscopy.

[28] Nieto J., Casas D. (2019). Salvage Hybrid APC After Failed Radiofrequency Ablation and Cryotherapy for Barrett’s Esophagus. Am. J. Gastroenterol..

[29] Orman E.S., Li N., Shaheen N.J. (2013). Efficacy and Durability of Radiofrequency Ablation for Barrett’s Esophagus: Systematic Review and Meta-Analysis. Clin. Gastroenterol. Hepatol..

[30] Tariq R., Enslin S., Hayat M., Kaul V. (2020). Efficacy of Cryotherapy as a Primary Endoscopic Ablation Modality for Dysplastic Barrett’s Esophagus and Early Esophageal Neoplasia: A Systematic Review and Meta-Analysis. Cancer Control.

[31] Guthikonda A., Cotton C.C., Madanick R.D., Spacek M.B., Moist S.E., Ferrell K., Dellon E.S., Shaheen N.J. (2017). Clinical Outcomes Following Recurrence of Intestinal Metaplasia After Successful Treatment of Barrett’s Esophagus with Radiofrequency Ablation. Am. J. Gastroenterol..

[32] Krishnamoorthi R., Singh S., Ragunathan K., Katzka D.A., Wang K.K., Iyer P.G. (2016). Risk of Recurrence of Barrett’s Esophagus after Successful Endoscopic Therapy. Gastrointest. Endosc..

[33] Hamade N., Desai M., Thoguluva Chandrasekar V., Chalhoub J., Patel M., Duvvuri A., Gorrepati V.S., Jegadeesan R., Choudhary A., Sathyamurthy A. (2019). Efficacy of Cryotherapy as First Line Therapy in Patients with Barrett’s Neoplasia: A Systematic Review and Pooled Analysis. Dis. Esophagus.

[34] Mohan B.P., Krishnamoorthi R., Ponnada S., Shakhatreh M., Jayaraj M., Garg R., Law J., Larsen M., Irani S., Ross A. (2019). Liquid Nitrogen Spray Cryotherapy in Treatment of Barrett’s Esophagus, Where Do We Stand? A Systematic Review and Meta-Analysis. Dis. Esophagus.

[35] Wronska E., Polkowski M., Orlowska J., Mroz A., Wieszczy P., Regula J. (2021). Argon Plasma Coagulation for Barrett’s Esophagus with Low-Grade Dysplasia: A Randomized Trial with Long-Term Follow-up on the Impact of Power Setting and Proton Pump Inhibitor Dose. Endoscopy.

[36] Estevinho M.M., Pinho R. (2022). Hybrid Argon Plasma Coagulation after Endoscopic Mucosal Resection—Some Caveats in the Comparison with Snare Tip Soft Coagulation. Gastrointest. Endosc..

