# Supplementary material for: Hybrid Argon Plasma Coagulation for Barrett’s Esophagus and for Colonic Mucosal Resection—A Systematic Review and Meta-Analysis
Source: Biomedicines. 2023 Apr 10;11(4):1139. doi: 10.3390/biomedicines11041139 (PMC10135954; doi:10.3390/biomedicines11041139)
Supplement: Supplementary file 1 [file biomedicines-11-01139-s001.zip › biomedicines-2299531-supplementary.pdf]

Supplementary Materials

Supplementary Table S1. Search strategy (date of last access 15th December 2022).

Search engine	Terms and keywords	Results
PubMed	"(hybrid argon plasma coagulation OR argon plasma coagulation) AND (colon OR endoscopic mucosal resection OR Barrett esophagus)"	419
Scopus	"(argon AND plasma AND coagulation) AND (Barrett AND esophagus OR colon)"	526
Web of Science	"hybrid argon plasma coagulation"	25
Cochrane Central Register of Controlled Trials (CENTRAL)	"hybrid argon plasma coagulation"	9

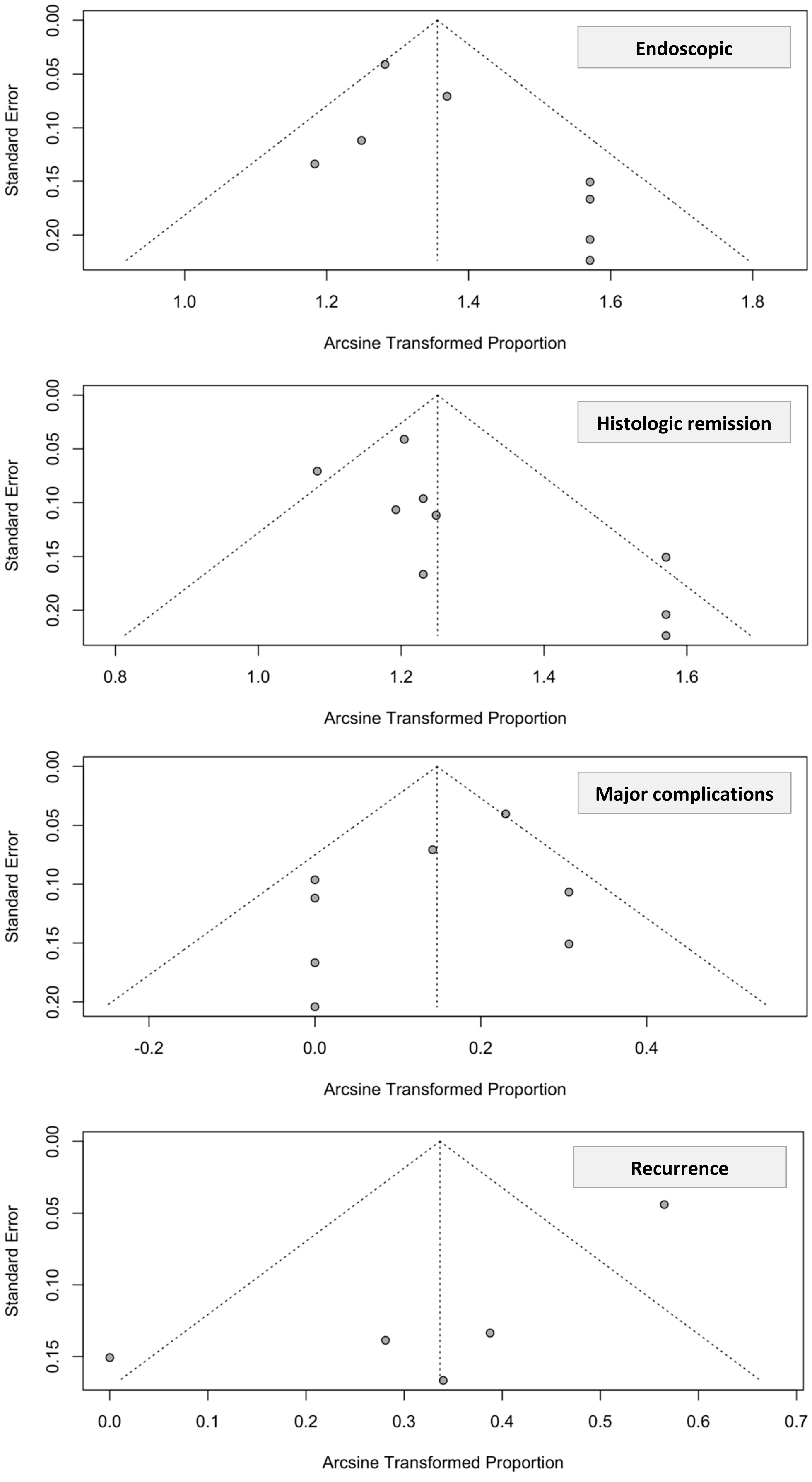

Supplementary Figure S1. Funnel plots for the proportions of outcomes' achievement after hybrid argon plasma coagulation (hAPC) for Barrett esophagus (BE) ablation.

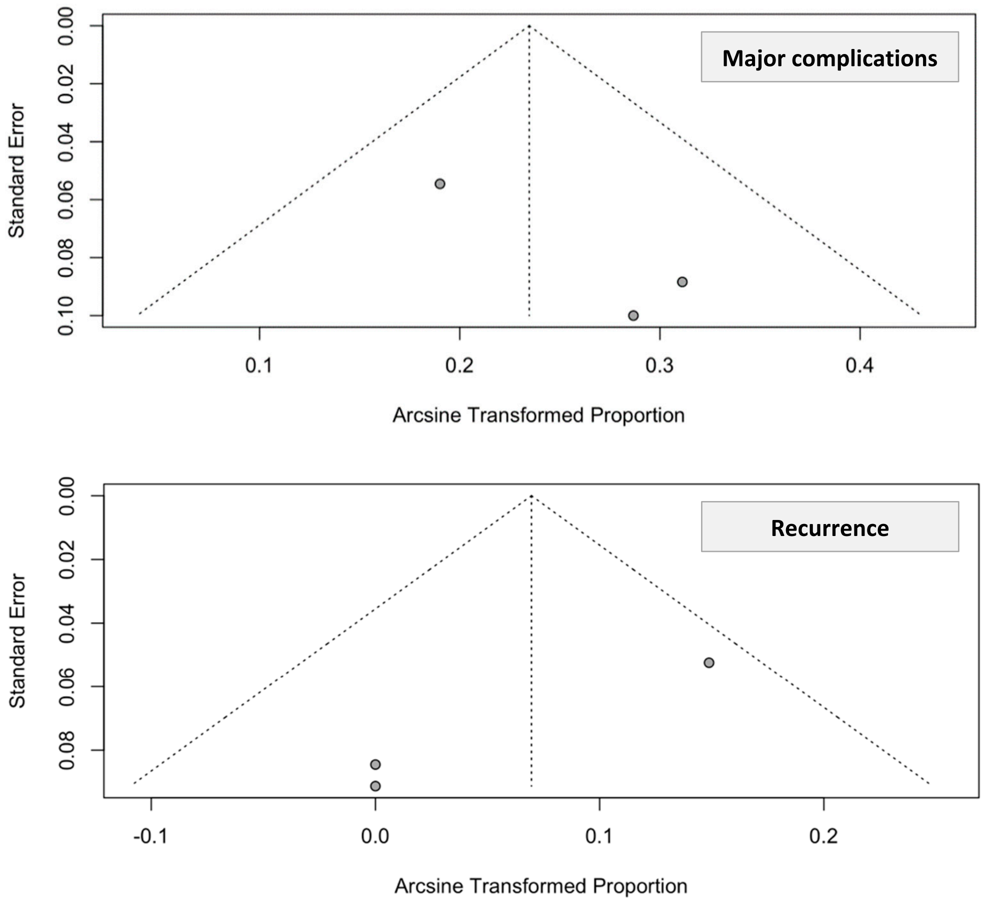

Supplementary Figure S2. Funnel plots for the proportions of outcomes' achievement after hybrid argon plasma coagulation (hAPC)-assisted endoscopic mucosal resection (EMR) of colonic lesions with more than 20 mm.

	CASP criteria												
**Study**	1	2	3	4	5a	5b	6a	6b	7	8	9	10	11
Knabe et al., 2022	Yes	Yes	Yes	Yes	Can’t tell	Yes	Yes	Yes	Yes	Yes	Yes	Yes	Yes
Levenick et al., 2022	Yes	Yes	Can’t tell	Yes	Can’t tell	Can’t tell	Yes	Can’t tell	Yes	Yes	Can’t tell	Yes	Yes
Morales Martínez et al., 2022	Yes	Can’t tell	Can’t tell	Yes	Can’t tell	No	Can’t tell	Yes	Yes	Yes	Can’t tell	Yes	Yes
Motchum et al., 2022	Yes	Yes	Yes	Yes	Can’t tell	Can’t tell	Yes	Can’t tell	Yes	Yes	Yes	Yes	Yes
Motz et al., 2022	Yes	Yes	Yes	Yes	Yes	Can’t tell	Yes	Can’t tell	Yes	Yes	Yes	Yes	Yes
Armenteros Torres, 2021	Yes	Can’t tell	Can’t tell	Yes	Can’t tell	No	Can’t tell	Can’t tell	Yes	Yes	Can’t tell	Yes	Yes
Kashin et al., 2021	Yes	Can’t tell	Can’t tell	Can’t tell	Can’t tell	Can’t tell	Can’t tell	Yes	Yes	Yes	Can’t tell	Yes	Yes
Shimizu et al., 2021	Yes	Yes	Can’t tell	Yes	Yes	Can’t tell	Yes	No	Yes	Yes	Can’t tell	Yes	Yes
Staudenmann et al., 2021	Yes	Yes	Can’t tell	Yes	Yes	Can’t tell	Yes	Yes	Yes	Yes	Can’t tell	Yes	Yes
Linn et al., 2020	Yes	Can’t tell	Can’t tell	Can’t tell	Can’t tell	Can’t tell	Can’t tell	Yes	Yes	Yes	Can’t tell	Yes	Yes
Trindade et al., 2020	Yes	Can’t tell	Can’t tell	No	Can’t tell	Can’t tell	No	No	Can’t tell	Yes	No	Yes	Yes
Nieto and Casas, 2019	Yes	Can’t tell	Can’t tell	Can’t tell	Can’t tell	Can’t tell	Can’t tell	Yes	Yes	Yes	Can’t tell	Yes	Yes
Manner et al., 2016	Yes	Yes	Can’t tell	Yes	Yes	Can’t tell	Yes	No	Yes	Yes	Can’t tell	Yes	Yes

● Yes | ● Can’t tell | ● No

Supplementary Figure S3. Results of the reporting quality analysis, using the Critical Appraisal Skills Programme (CASP) checklist. Checklist criteria: 1. Did the study address a clearly focused issue?; 2. Was the cohort recruited in an acceptable way?; 3. Was the exposure accurately measured to minimize bias?; 4.Was the outcome accurately measured to minimize bias?; 5a. Have the authors identified all important confounding factors?; 5b. Have they take account of the confounding factors in the design and/or analysis?; 6a. Was the follow up of subjects complete enough?; 6b. Was the follow up of subjects long enough? 7. Are the results precise?; 8. Do you believe the results?; 9. Can the results be applied to the local population?; 10. Do the results of this study fit with other available evidence?; 11. Does this study have practical implications?
